# Separation and Determination of Small Quantities of Aluminum in Steel

**DOI:** 10.6028/jres.064A.024

**Published:** 1960-06-01

**Authors:** Bruce B. Bendigo, Rosemond K. Bell

## Abstract

A method is described for determining small amounts of aluminum (0.01 to 0.3 percent) in stainless and carbon steels. A perchloric-sulfuric acid solution of the steel is electrolyzed in a mercury cathode cell to remove most of the iron, and an extraction with chloroform is made to remove elements such as aluminum, residual iron, and titanium as cupferrates from a solution buffered at *p*H 3.5. These elements are converted from cupferrates to perchlorates; all except aluminum are then extracted as cupferrates with chloroform from 4 *N* hydrochloric acid. Aluminum in the acid solution is determined photometrically with aluminon (ammonium aurintricarboxylate) at a wave length of approximately 540 millimicrons. An accuracy of ± 0.005 percent aluminum is indicated.

## 1. Introduction

Steel may contain up to 1 percent or more of aluminum as an alloying agent, or it may contain less than 0.1 percent aluminum introduced either as a final deoxiding agent or from the processing materials.

The determination of aluminum in steel usually requires the separation of this element from major amounts of iron, as well as from certain other elements that would interfere in the final estimation of the aluminum by photometric methods such as the aluminon method [4].^1^

A number of procedures have been used for this initial concentration step, for example, hydrolytic precipitation with sodium bicarbonate or sodium hydroxide, extraction of iron with ether [13], 8-hydroxyquinoline precipitation in cyanide solution [1], electrolysis in a mercury cathode cell [3], extraction of iron cupferrate [2], and separation by ion exchange [6, 7].

Consideration of the relative advantages and limitations of the above-mentioned procedures, as well as preliminary experimental work, led to the selection of the electrolytic separation as best suited for the initial separation step in the procedure described in this paper.

In the method to be described, the sample of steel is dissolved in hydrochloric acid, perchloric acid is added, and the solution is evaporated to fumes of perchloric acid. The acid solution is electrolyzed in a mercury cathode cell to remove most of the iron. Aluminum, residual iron, and certain other elements not removed by electrolysis are extracted as cupferrates from the electrolyte at *p*H 3.5 with chloroform. These elements are converted from cupferrates to perchlorates; all except aluminum are then extracted as cupferrates with chloroform from 4 *N* hydrochloric acid. Aluminum in the aqueous portion is then determined photometrically with aluminon. Two days are usually required to complete a determination.

## 2. Apparatus and Reagents

### Mercury Cathode Cell

A Dynacath type of mercury cathode cell was used to electrolyze the solutions.

### Colorimeter

An Evelyn photometer, with voltage stabilizer and galvanometer, was used for the quantitative optical measurements. Matched test tubes (22 by 175 mm) were used as absorption cells.

### Cupferron Solution (60 g/liter)

Dissolve 6 g of cupferron in 100 ml of cold water and filter. Prepare fresh as needed.

### Thioglycolic Acid Solution (100 ml/liter)

Dilute 10 ml of thioglycolic acid to 100 ml with water and filter, if necessary. This solution is stable for 1 week.

### Aluminon-Buffer Composite Solution

Dissolve 125 g of ammonium acetate in 250 ml of water and add 20 ml of glacial acetic acid. Filter through a tight paper. While stirring this filtrate well, add first a solution of 0.250 g of aluminon (ammonium aurintricarboxylate) dissolved in 50 ml of water, followed by a solution of 0.5 g of benzoic acid dissolved in 20 ml of methanol. Dilute the resulting solution to 500 ml, add 250 ml of glycerol, and stir well. Store in a dark glass-stoppered bottle.

### Standard Aluminum Solution A (1 ml=1.00 mg aluminum)

Transfer 1.000 g of high-purity aluminum to a 1-liter volumetric flask. Add 50 ml of hydrochloric acid (1 + 1) 2 and heat gently until the metal is completely dissolved. Cool to room temperature, dilute to the mark, and mix.

### Standard Aluminum Solution B (1 ml=0.05 mg aluminum)

Transfer exactly 50 ml of the standard aluminum solution A to a 1-liter volumetric flask. Add 10 ml of perchloric acid (70 to 72%), dilute to the mark, and mix. This solution should be prepared as needed.

### Dinitrophenol Indicator (0.2 g/liter)

Dissolve 20 mg of 2,4-dinitrophenol in 100 ml of water.

## 3. Procedure

Transfer 5 g of the steel sample (0.01 to 0.3 percent aluminum) to a 600-ml beaker and add 75 ml of hydrochloric acid (2+1). Heat gently until the sample is decomposed. Carefully add nitric acid (1+1) to oxidize the iron and add 20 ml in excess. Cool and add 50 ml of perchloric acid (70 to 72%). Evaporate the solution to fumes of perchloric acid, cover the beaker, and continue the heating for 10 min. If the chromium exceeds 5 percent, add hydrochloric acid in small portions to the hot perchloric acid solution until no more chromyl chloride is volatilized. Cool, add 200 ml of water, and boil for several minutes. Remove from the heat, add paper pulp, and let stand until the precipitate settles. Filter through a close-textured paper containing paper pulp. Wash 10 times with sulfuric acid (1+99) and reserve the filtrate.

Transfer the paper and contents to a 30-ml platinum crucible. Char the paper and ignite under good oxidizing conditions at 500° C until the carbon is destroyed. Cool, add 2 drops of sulfuric acid (1+1), and 5 to 10 ml of hydrofluoric acid (48 percent) to the ignited residue. Heat the crucible in an air bath until the acids are expelled. Cool the crucible, and fuse the residue with 1 g of fused sodium bisulfate. Cool, add 20 ml of sulfuric acid (1+99) to the crucible, and heat gently until the melt is disintegrated. Transfer the contents to a 150-ml beaker with sulfuric acid (1+99). Boil for 5 min to insure complete solution of the aluminum salts and filter through a close-textured paper. Wash 5 times with sulfuric acid (1+99) and discard the paper and contents. Cool the filtrate to room temperature and combine it with the reserved filtrate. Transfer the combined filtrates to a 500-ml volumetric flash, dilute to the mark, and mix.

Transfer a 100-ml aliquot portion of the solution (this portion is equivalent to 1 g of the sample) with a pipet to a mercury cathode cell containing 35 ml of mercury. Add 5 ml of sulfuric acid (1+1) and electrolyze with a current of 15 amp until the solution is colorless. About 15 min are required to remove 1 g of iron. Remove the electrolyte from the cell and wash the electrodes and cell with sulfuric acid (1+99). Filter the electrolyte and washings through a medium-textured paper. Wash 5 times with sulfuric acid (1+99) and discard the paper. Evaporate the filtrate to about 50 ml, then cool.

Neutralize the solution with ammonium hydroxide, using litmus as an indicator. Immediately acidify with hydrochloric acid (1+1) and add 10 ml in excess. Cool to room temperature and add ammonium hydroxide until the *p*H is between 3 and 7, as indicated by a *p*H meter. Immediately add 10 ml of formic acid, and mix. Add ammonium hydroxide until the *p*H is 3.5±0.1.

Transfer the buffered solution to a 250-ml separatory funnel and cool in ice water for 15 min. Disregard any insoluble salts. Add 25 ml of cupferron solution, mix, and allow to stand for 15 min in ice water. Extract the cupferrates with 25-ml portions of chloroform until the chloroform layer remains colorless. Allow the layers to separate while the funnel cools in ice water. Transfer the chloroform layers to a 300-ml Kjeldahl flask containing 10 ml of water. Three or four extractions are usually sufficient. Discard the aqueous layer.

Heat the Kjeldahl flask in a water bath until the chloroform evaporates. Cool and add 10 ml of nitric acid and 6 ml of perchloric acid (70 to 72%). Swirl the solution over a free flame until the perchloric acid condenses at the mouth of the flask. Cool, add 10 ml of hydrochloric acid, and heat as directed before until perchloric acid condenses in the neck of the flask. Cool, add 50 ml of water, and boil for 5 min. Cool to room temperature.

Transfer the solution to a 250-ml separatory funnel, wash the flask with 60 ml of hydrochloric acid (1+1), and add the washings to the funnel. Dilute the resulting solution to 100 ml, mix, and cool the funnel in ice water for 15 min. Add 10 ml of the cupferron solution, mix, and allow to stand in the ice water for 10 min. Extract the cupferrates with one 25-ml portion and several 10-ml portions of chloroform until the chloroform layer remains colorless. Allow the layers to separate while the funnel cools in ice water. Discard the chloroform extracts. Transfer the aqueous portion to the same Kjeldahl flask used before.

Add 10 ml of nitric acid (1+1) and evaporate the solution to about 25 ml. Add 10 ml of nitric acid and continue the evaporation until the perchloric acid condenses at the top of the flask. Cool, add 50 ml of water, and boil for 5 min. Cool to room temperature.

Transfer the solution to a 200-ml volumetric flask, dilute to the mark, and mix. Transfer a 20-ml aliquot portion with a pipet to a 100-ml volumetric flask. Add 3 drops of the dinitrophenol indicator solution to the flask and neutralize the solution by dropwise addition of ammonium hydroxide until the indicator turns yellow. Immediately add hydrochloric acid (1+1) dropwise until the solution is colorless and then add 1 drop in excess. Add 2 ml of the thioglycolic acid solution and exactly 15.0 ml of the aluminon-buffer composite solution. Because the composite solution is rather viscous, the pipet should drain for a minute or two. Place the flask in a 400-ml beaker containing boiling water and heat for exactly 4 min while maintaining the boiling temperature. Remove from the heat and let stand for 1 min. Cool in running water for 4 min. Dilute to the mark and mix. Transfer a suitable portion of the solution to an absorption cell and measure the absorbancy or transmittancy at 540 m*μ*, using water as a reference solution. Obtain from a calibration curve the milligrams of aluminum in the final solution. Correct the value obtained for a reagent blank carried through all steps of the procedure.

## 4. Preparation of the Calibration Curve

To eight 100-ml volumetric flasks transfer 0.0, 0.5, 1.0, 2.0, 3.0, 4.0, 5.0, and 6.0 ml of the standard aluminum solution B. Add 5 drops of perchloric acid (70 to 72%) to each and dilute to 20 ml. Add 3 drops of dinitrophenol indicator to each flask, and follow with dropwise addition of ammonium hydroxide until the indicator turns yellow. Immediately add hydrochloric acid (1+1) dropwise until the solution is colorless and add 1 drop in excess. Add 2 ml of the thioglycolic acid solution and 15.0 ml of the aluminon-buffer composite solution. Place each flask in a 400-ml beaker containing boiling water. Heat for exactly 4 min while maintaining the boiling temperature. Remove from the heat and let stand for 1 min. Cool in running water for 4 min, dilute to the mark, and mix. Measure the absorbancy or transmittaney of each solution as directed for the analysis sample. Plot the values obtained against milligrams of aluminum per 100 ml of solution.

## 5. Results

The data in table 1 show the results obtained when the recommended procedure is used to determine aluminum in solutions of 1 g of stainless steel (having less than 0.001 percent aluminum) containing known additions of aluminum. The data in table 2 indicate the results obtained by applying the recommended procedure to various NBS standard samples.

## 6. Discussion

After electrolysis in the mercury cathode cell, the electrolyte contains all of the aluminum, titanium, vanadium, zirconium, phosphorus, alkaline earths, and residual iron and chromium. It will also contain the earth acids and tungsten which have escaped filtration, as well as relatively large amounts of perchloric acid. Further separations of aluminum are based on the fact that aluminum cupferrate is extracted by chloroform from a solution buffered between *p*H 2 and 5 [5], but not from a solution 4 *N* in acid [11, 14].

There are several advantages gained by an extraction of cupferrates from the electrolyte buffered at *p*H 3.5. At this *p*H, aluminum is separated from phosphate. This is of possible utility when aluminum is determined as the oxide after precipitation as the hydroxide. At *p*H 3.5, the cupferron separation of aluminum removes the relatively large amounts of perchloric and sulfuric acids which would interfere by forming ammonium salts later in the photometric determination of the aluminum. Most of the residual chromium in the electrolyte is also removed in the aqueous phase. Aluminum cupferrate is completely extracted by chloroform from a solution that is buffered with formic acid to *p*H 3.5. Measured quantities of aluminum were added to and extracted from this buffered solution, and very little, if any, of the added aluminum was lost, as can be seen from the results shown in table 3.

The chloroform extraction of cupferrates from a 4 *N* hydrochloric acid separates interfering elements from the aluminum, which remains in the aqueous portion. Very small amounts of chromium may accompany the aluminum. However, tests showed that 0.15 mg of chromium did not interfere in the photometric determination of the aluminum with aluminon. After the small amount of unextracted cupferron has been destroyed, the aluminum can be determined by any suitable method. When aluminum is present in small quantities, it is determined conveniently by the aluminon-photometric method. When larger amounts of aluminum are present, it can be determined gravimetrically, if desired. Tests were made for aluminum remaining in the aqueous solution after extraction with chloroform from a 4 *N* acid solution to which cupferron and known quantities of aluminum had been added. These results are given in table 4.

It is well established that iron can be separated from aluminum by electrolysis in a mercury cathode cell [8, 9]. Wiberley and Bassett [16] suggests a one-normal mixture of perchloric-sulfuric acids as the electrolyte. It is not necessary to completely remove the iron because small remaining quantities are separated later in the recommended procedure. Because chromium is removed slowly by electrolysis, it is advantageous, with steels containing more than 5 percent chromium, to remove most of the chromium as chromyl chloride prior to electrolysis. No significant errors occur if less than 50 mg of chromium remain before electrolysis. No aluminum is lost during electrolysis, according to Scherrer and Mogerman [12]. When known quantities of aluminum were added to solutions representative of those present after electrolysis, and the recommended procedure followed, quantitative results were obtained, as shown by the data in table 5.

The recommended aluminon photometric procedure has been described by Pellowe and Hardy [10]. The optimum amount of aluminum in the final aliquot is between 0.02 and 0.30 mg. It should be noted that the color of the solution has a significant temperature coefficient [15]. Therefore, when absorbancy measurements are made, the temperature of test solutions and standards should not differ by more than 5° C.

As described, the method recommended in this paper is used to determine *total* aluminum. If desired, however, the acid-insoluble aluminum can be separated from that which is soluble by filtering the hydrochloric acid solution of the sample; each can then be determined separately in the solution and residue.

The recommended sample size of 5 g will minimize the error due to the segregation of aluminum in the alloy. In the procedure described, an aliquot equivalent to 1 g of steel is taken for electrolysis.

## Figures and Tables

**Table 1:** Results obtained for aluminum by applying the recommended procedure to solutions of one gram of a stainless steel (*Al* <0.001%) containing known additions of aluminum

Added	Found^a^	Difference
		
*mg*	*mg*	*mg*
0.02	0.02	0.0
.04	.04	.0
.06	.06	.0
.10	.09	−.01
.10	.09	−.01
.10	.10	.0
.10	.11	+.01
.20	.21	+.01
.20	.21	+.01
.30	.29	−.01
.30	.32	+.02
.50	.47	−.03
.80	.77	−.03
1.10	1.08	−.02

a Corrected for blank on reagents plus the steel.

**Table 2:** Results obtained for aluminum by applying the recommended procedure to various National Bureau of Standards standard steels

NBS standard steel	Found	Certificate value	Type of steel
			
	%	%	
1164	0.0043	a0.005	Si 0.5, V 0.3.
	.0048		
1166	.014	a.015	Open-Hearth Iron (Ti 0.06).
	.015		
1162	.022	a.023	Ni 0.7, Cr 0.7, Sn 0.07, Ti 0.04, Nb 0.1, Zr 0.06.
	.021		
111b	.042	.042	Ni 1.8, Mo 0.3.
	.044		
65d	.056	.059	Basic Electric Steel (C 0.3).
	.056		
	.055		
	.057		
	.058		
22c	.110	.116	Bessemer Steel.
	.114		
1167	.156	a.16	Ti 0.3, W 0.2, Zr 0.1, Nb 0.3, Ta 0.2.
	.150		
1165	.185	a.19	Open-Hearth Iron (Ti 0.2).
	.187		

a Provisional value.

**Table 3:** Results obtained for aluminum when aluminum cupferrate is extracted with chloroform from a solution buffered at pH 3.5 with formic acid

Added	Found	Difference
		
*mg*	*mg*	*mg*
0.27	0.26	−0.01
.50	.49	−.01
.73	.73	.00
1.03	1.01	−.02

**Table 4:** Results obtained for aluminum in the aqueous solution after a 4 N *HCl*-*HClO*_4_ solution containing aluminum and excess cupferron is extracted with chloroform

Added	Found	Difference
		
*mg*	*mg*	*mg*
0.02	0.03	+0.01
.04	.04	.00
.06	.06	.00
.08	.08	.00

**Table 5:** Results obtained for aluminum in synthetic solutions representative of the electrolyte remaining after electrolysis with a mercury cathode cell

Aluminum			Composition of solutions			
Added	Found	Difference	Iron	Chromium	Titanium	Vanadium
						
*mg*	*mg*	*mg*	*mg*	*mg*	*mg*	*mg*
0.25	0.26	+0.01	1	1	0.5	0.5
.50	.50	.00	1	1	.5	.5
.75	.72	−.03	1	1	.5	.5

## Footnotes

1 Figures in brackets indicate the literature references at the end of this paper.

2 Hydrochloric acid (1+1) denotes 1 volume of concentrated hydrochloric acid, sp gr 1.18, diluted with 1 volume of water. Nitric acid (1+1) denotes 1 volume of nitric acid, sp gr 1.42, diluted with 1 volume of water. Sulfuric acid (1+1) denotes 1 volume of sulfuric acid, sp gr 1.84, diluted with 1 volume of water. If no dilution is specified, the concentrated reagent is meant.
